# Supplementary material for: Modelling the Cost-Effectiveness of Implementing a Dietary Intervention in Renal Transplant Recipients
Source: Nutrients. 2021 Apr 2;13(4):1175. doi: 10.3390/nu13041175 (PMC8066697; doi:10.3390/nu13041175)
Supplement: Supplementary file 1 [file nutrients-13-01175-s001.pdf]

- **Supplementary Material**

Table S1. Parameters for DWFG and renal graft failure distribution.

Variable	Value	Range	Source
**DWFG**	**Weibull parameters**	**SE**	**Source**
**Group 1 potassium**	Intercept = 2.622	0.112	UMCG dataset [18]
	Logscale = -0.274	0.077	
**Group 2 potassium**	Intercept = 2.977	0.148	
	Logscale = -0.274	0.077	
**Group 3 potassium**	Intercept = 3.086	0.153	
	Logscale = -0.274	0.077	
**Group 1 DASH**	Intercept = 2.708	0.127	
	Logscale = -0.309	0.083	
**Group 2 DASH**	Intercept = 2.946	0.152	
	Logscale = -0.309	0.083	
**Group 3 DASH**	Intercept = 2.925	0.159	
	Logscale = -0.309	0.083	
**GF**			UMCG dataset [18]
**Group 1 potassium**	Intercept = 3.201	0.194	
	Logscale = -0.126	0.097	
**Group 2 potassium**	Intercept = 3.630	0.217	
	Logscale = -0.126	0.097	
**Group 3 potassium**	Intercept = 3.824	0.233	
	Logscale = -0.126	0.097	
**Group 1 DASH**	Intercept = 3.125	0.193	
	Logscale = -0.149	0.103	
**Group 2 DASH**	Intercept = 3.692	0.022	
	Logscale = -0.149	0.103	
**Group 3 DASH**	Intercept = 3.808	0.253	
	Logscale = -0.149	0.103	

Abbreviations: DASH, Dietary Approach to Stop Hypertension; DWFG, death with a functioning graft; GF, graft failure; SE, standard error; UMCG, University Medical Centre Groningen.

Table S2. Discounted incremental and total costs for the DASH per cost category.

Cost category	DASH no dietary intervention	DASH with dietary intervention	Incremental costs
Functioning graft upkeep	€ 88,306,866	€ 109,296,474	€ 20,989,608
Graft failure upkeep	€ 118,704,311	€ 71,023,249	€ -47,681,062

Transplantation costs	€ 8,251,452	€ 4,855,403	€ -3,396,049
Graft failure costs	€ 760,403	€ 470,717	€ -289,685
Productivity costs	€ 3,273,004	€ 2,495,345	€ -777,659
Intervention costs	€ -	€ 515,140	€ 515,140
Extra dietary costs	€ -	€ 2,766,562	€ 2,766,562
Death	€ 724,190	€ 662,550	€ -61,640
Total	€ 220,020,226	€ 192,085,440	€ -27,934,786

Abbreviations: DASH, Dietary Approach to Stop Hypertension

Table S3. Discounted incremental and total costs for potassium supplementation per cost category.

Cost category	Potassium supplementation no dietary intervention	Potassium supplementation with dietary intervention	Incremental costs
Functioning graft upkeep	€ 84,747,240	€ 113,326,587	€ 28,579,347
Graft failure upkeep	€ 106,066,982	€ 77,892,517	€ -28,174,464
Transplantation costs	€ 7,546,240	€ 5,240,387	€ -2,305,853
Graft failure costs	€ 679,994	€ 516,376	€ -163,619
Productivity costs	€ 3,351,790	€ 2,437,692	€ -914,098
Intervention costs	€ -	€ 515,140	€ 515,140
Extra dietary costs	€ -	€ 1,348,066	€ 1,348,066
Death	€ 744,970	€ 642,648	€ -102,321
Total	€ 203,137,216	€ 201,919,413	€ 1,217,803
